# The Challenge of Deep Learning for the Prevention and Automatic Diagnosis of Breast Cancer: A Systematic Review

**DOI:** 10.3390/diagnostics14242896

**Published:** 2024-12-23

**Authors:** Jhelly-Reynaluz Pérez-Núñez, Ciro Rodríguez, Luis-Javier Vásquez-Serpa, Carlos Navarro

**Affiliations:** Facultad de Ingeniería de Sistemas e Informática, Universidad Nacional Mayor de San Marcos (UNMSM), Lima 15081, Peru; crodriguezro@unmsm.edu.pe (C.R.); luis.vasquez2@unmsm.edu.pe (L.-J.V.-S.); cnavarrod@unmsm.edu.pe (C.N.)

**Keywords:** breast cancer, deep learning, convolutional neural network, transfer learning network

## Abstract

Objectives: This review aims to evaluate several convolutional neural network (CNN) models applied to breast cancer detection, to identify and categorize CNN variants in recent studies, and to analyze their specific strengths, limitations, and challenges. Methods: Using PRISMA methodology, this review examines studies that focus on deep learning techniques, specifically CNN, for breast cancer detection. Inclusion criteria encompassed studies from the past five years, with duplicates and those unrelated to breast cancer excluded. A total of 62 articles from the IEEE, SCOPUS, and PubMed databases were analyzed, exploring CNN architectures and their applicability in detecting this pathology. Results: The review found that CNN models with advanced architecture and greater depth exhibit high accuracy and sensitivity in image processing and feature extraction for breast cancer detection. CNN variants that integrate transfer learning proved particularly effective, allowing the use of pre-trained models with less training data required. However, challenges include the need for large, labeled datasets and significant computational resources. Conclusions: CNNs represent a promising tool in breast cancer detection, although future research should aim to create models that are more resource-efficient and maintain accuracy while reducing data requirements, thus improving clinical applicability.

## 1. Introduction

Breast cancer (BC) is a dangerous condition that develops when the cells of the mammary gland tissue grow abnormally and uncontrollably, resulting in tumors [1]. Breast cancer was first mentioned in the Edwin Smith Papyrus, dated between 3000 and 2500 BC [2]. A breakthrough in treating this disease occurred in 1882 when William Halsted performed the first mastectomy.

Breast cancer is among the biggest causes of death among women globally, with its incidence and fatality rates fluctuating based on geographic location [3]. The prevalence and deadliness of this disease differ across various regions around the world, making it a significant public health concern for women worldwide [4].

Despite advancements in medical imaging and treatment, disparities in diagnostic accuracy and access to early intervention persist, particularly in low-resource regions. In 2020, a total of 2.3 million women were diagnosed with breast cancer, and 29.8% of them lost their lives due to the disease [5]. By 2022, breast cancer diagnoses continued to rise, underscoring the urgent need for improved diagnostic tools and accessible treatments.

The advent of artificial intelligence (AI) and machine learning (ML) has brought renewed hope for improving BC diagnosis, offering tools with the potential to enhance diagnostic accuracy and efficiency through non-invasive imaging analysis. Deep learning, a branch of ML, is at the forefront of these innovations, especially in its capacity to analyze medical images such as mammograms, ultrasounds, and thermograms [6,7]. These technologies can reveal patterns in imaging data that may go unnoticed by human practitioners, enabling earlier detection and classification of lesions as benign or malignant.

This paper systematically reviews the application of ML, especially deep learning techniques, in detecting and classifying breast cancer. It aims to bridge existing research gaps by analyzing key areas where ML significantly contributes to BC detection, focusing on identifying malignant and benign lesions. Furthermore, this review evaluates the current maturity and effectiveness of ML-based diagnostic tools in breast cancer detection, comparing them with conventional diagnostic methods and assessing factors such as data quality and model robustness [8,9].

Finally, the review offers directions for future research, proposing the development of adaptable, ethically guided ML models that can be applied effectively in resource-constrained environments. By addressing these needs, ML advancements can potentially enhance diagnostic accuracy and accessibility, ultimately improving BC health outcomes and reducing global mortality rates.

## 2. Diagnostic Imaging Modalities in Breast Cancer

Effective breast cancer screening involves multiple diagnostic modalities, each serving a unique role in identifying abnormalities in breast tissue. Alongside imaging techniques, patient awareness and thorough clinical evaluations are vital in breast cancer detection and management.

**Medical Consultation and Self-Examination**

According to Segnan [10], a complete medical consultation includes a detailed patient history to assess risk factors and rule out breast-related symptoms. This is typically followed by a physical examination and targeted questions aimed at distinguishing breast cancer from other conditions, such as fibroadenomas or cysts. Garcia et al. [11] emphasize the importance of women being familiar with their breasts’ appearance for early detection of any irregularities, though some guidelines question the effectiveness of monthly self-exams. Picazo et al. [12] also support self-exam value as a potential tool for early awareness.

**Mammography:** This is the primary imaging technique for breast cancer screening, utilizing X-ray technology to create detailed images of breast tissue. The sensitivity of mammography, around 67.8%, can vary depending on age, ethnicity, and individual factors. It is important to note that the effectiveness of mammography can be affected by the operator’s skills and the equipment’s functionality [13]. Mammography is especially recommended for women aged 40 and above, as it allows for the early detection of tumors before they become symptomatic.

**Ultrasound:** Ultrasound is often used as an adjunct to mammography, particularly in younger women, pregnant women, those with dense breasts, and women with breast implants. This non-invasive technique provides a specificity of 98% but is highly operator-dependent, which can influence diagnostic accuracy. Ultrasound is especially useful in differentiating between solid and cystic masses, making it valuable in distinguishing benign from malignant lesions [11].

**Magnetic Resonance Imaging (MRI):** This is recommended for women at a high risk of breast cancer, particularly those with genetic predispositions or a family history of the disease. MRI offers a sensitivity range of 70% to 96% and a specificity of 67% to 100%, making it particularly useful for detecting tumors in dense breast tissue. This technique provides detailed imaging that can reveal structural abnormalities not easily seen by other methods.

**Computed Tomography (CT) and Positron Emission Tomography (PET):** CT and PET scans are generally reserved for more advanced cases to detect metastases or monitor response to treatment. CT scans provide a sensitivity of 91% and a specificity of 93%. PET scans are particularly effective for assessing treatment response, with a sensitivity of 61% and a specificity of 80%. These modalities are instrumental in staging and assessing the disease’s progression [14].

**Image-Guided Biopsy**

Biopsy, especially when guided by imaging techniques like mammography or ultrasound, is critical for confirming a breast cancer diagnosis. Among the different types of biopsies, fine needle aspiration involves extracting a small number of cells, while core needle biopsy collects larger tissue samples, often requiring local anesthesia. Image-guided biopsies play a crucial role in assessing cancer stage and prognosis and informing treatment options. This procedure provides comprehensive insights into the clinical status of the disease, enabling tailored therapeutic approaches based on accurate staging and prognostic evaluation [11,15].

Early detection and prompt treatment are critical for reducing breast cancer mortality [16]. Mammography plays an essential role in screening as it allows one to identify tumors before they are palpable or symptomatic, thus facilitating early treatment [17]. Table 1 presents guidelines from various medical, academic, and professional organizations regarding screening recommendations for women at a moderate and high risk of developing breast cancer.

**Figure 1:**
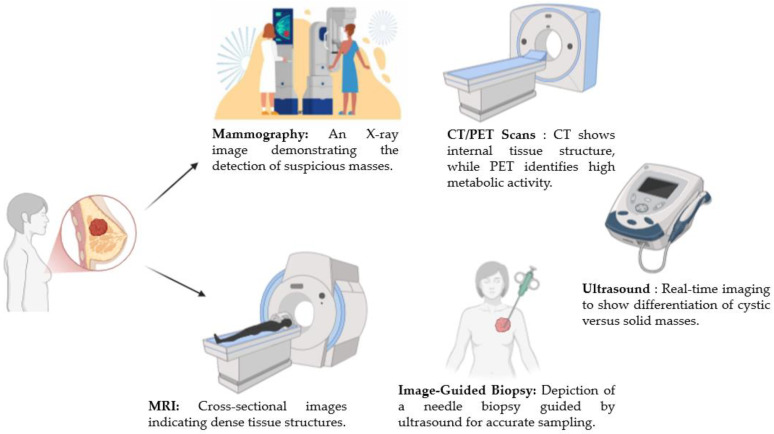
Overview of imaging modalities in breast cancer detection (created with BioRender.com).

Figure 1 displays various imaging techniques for breast cancer diagnosis, including mammography, ultrasound, CT/PET scans, MRI, and image-guided biopsy. Each modality offers unique diagnostic insights, aiding in the accurate detection, differentiation, and sampling of breast abnormalities.

## 3. Deep Learning Applications in Breast Cancer Detection

Recent advancements in deep learning algorithms have sparked a wave of optimism in breast cancer detection. The remarkable performance of these algorithms has inspired many researchers to explore their potential in diagnosing BC. This diagnostic aid system (DAS) approach, capable of distinguishing breast lumps as benign or malignant, involves a selection process, lesion segmentation, and image feature calculation [18]. The future of breast cancer detection looks promising, and it has the potential for deep learning.

The primary categories of deep learning-based BC diagnostic methods that are examined in this section include convolutional neural networks (CNNs), artificial neural networks (ANNs), autoencoders, Deep Belief Networks, Extreme Learning Machines (ELMs), and Generative Adversarial Networks (GANs).

**Artificial Neural Networks (ANNs):** In the context of breast cancer (BC) diagnosis, artificial neural networks (ANNs) have demonstrated potential as complementary tools to enhance diagnostic accuracy, especially within research and computational environments. Although ANNs are not yet a standard or essential component of clinical practice, advancements in deep learning suggest that these models can play a valuable role in analyzing medical images, identifying complex patterns, and differentiating between benign and malignant lesions [7,18,19]. However, the use of ANNs in clinical settings remains limited worldwide, with traditional diagnostic methods, such as physical examinations, mammography, and histology, continuing to be the primary techniques for breast cancer detection. ANNs with multiple hidden layers have proven to be effective at handling complex image classification tasks and capturing intricate details within breast tissue images, making them valuable for research applications [21]. Nevertheless, these models demand considerable computational resources and large datasets for training, and their performance can decline when managing high-dimensional data that lack adequate labeled examples. Moreover, the interpretability of deep learning models in clinical settings is a concern, as they often function as ’black boxes’, making it difficult for clinicians to understand the reasoning behind their decisions.

Currently, ANNs are predominantly applied in experimental and research-based diagnostic aid systems (DAS), presenting promising possibilities for future use in breast cancer diagnostics. These systems are capable of performing functions such as lesion segmentation, feature extraction, and classification, potentially serving as valuable complements to traditional diagnostic techniques and supporting clinicians in the accurate classification of tumors [20,22,23]. The potential integration of ANNs in clinical settings is an exciting prospect for the future of healthcare.

**Deep Belief Network (DBN):** DBNs are complex neural networks with multiple layers that use unsupervised pre-training techniques, such as the contrastive divergence algorithm, to learn features from unlabeled data. In breast cancer detection, DBNs help by extracting hierarchical features from mammograms and ultrasound images, capturing both low- and high-level patterns critical for identifying malignancies. This structure makes DBNs effective at improving classification accuracy and reducing noise, which are essential for accurate breast cancer diagnosis [24,25,26]. DBNs are effective due to their ability to perform unsupervised pre-training, which allows them to learn useful representations of unlabeled data and address large unlabeled datasets. Their hierarchical structure facilitates learning low-level and high-level features, improving data abstraction and representation. In addition, DBNs are efficient in dimensionality reduction, extracting relevant features, and minimizing noise, which improves performance in classification and regression tasks. Pre-training also provides good weight initialization, avoiding problems such as gradient vanishing and improving the efficiency of deep network training [27].

**Stochastic Generative Models:** Stochastic generative models, particularly Restricted Boltzmann Machines (RBMs), play a significant role in breast cancer imaging by performing dimensionality reduction and pre-training of unsupervised deep networks. RBMs are probabilistic models that capture interdependencies between visible and hidden layers, which allows them to learn meaningful representations from unstructured data. In breast cancer detection, RBMs help identify patterns within mammographic or histopathological images, aiding in the classification of breast tissue as benign or malignant [24,28]. Due to their ability to learn hierarchical feature representations, RBMs can improve the accuracy of diagnostic models, especially when labeled data are scarce. However, RBMs can face challenges in terms of scalability and convergence, which limits their effectiveness when handling large datasets typical in breast cancer research. Despite these limitations, RBMs remain valuable for pre-training layers in deep networks, making the networks more robust for breast cancer diagnostics [19].

**Autoencoder:** An autoencoder is an artificial neural network commonly used for data compression, feature extraction, and noise reduction in medical imaging, especially in breast cancer diagnostics. It consists of an encoder that compresses input data into a low-er-dimensional representation, capturing essential features, and a decoder that reconstructs the original data as accurately as possible. In breast cancer detection, autoencoders have shown effectiveness in tasks such as denoising mammograms and ultrasound images, facilitating better visualization of tumor characteristics [29].

Autoencoders have been particularly effective in improving the clarity of mammographic images, assisting in anomaly detection, and generating synthetic data for augmenting limited datasets, all of which contribute to higher accuracy in breast cancer detection models [30,31]. Furthermore, autoencoders are employed to generate synthetic data, which is valuable for augmenting limited training datasets, especially when labeled images are scarce. Studies demonstrate that autoencoders, particularly when combined with convolutional neural networks (CNNs), significantly improve breast cancer classification performance by providing a more comprehensive feature extraction process [31,32].

**Convolutional Neural Network (CNN):** Convolutional neural networks (CNNs) have become a cornerstone in detecting and classifying breast cancer due to their unique architecture and ability to handle image data effectively. CNNs are built upon three primary design principles: weight sharing, which reduces the number of parameters in the network; subsampling (or pooling), which reduces the spatial dimensions of feature maps while retaining important features; and local receptive fields, which allows the network to focus on localized regions of an image. A typical CNN is composed of three types of layers: convolutional layers, pooling layers, and fully connected output layers [19,33].

For breast cancer detection, CNNs are highly effective at extracting features directly from medical images, such as mammograms, and performing classification in a single architecture. However, CNNs require many labeled images to achieve high accuracy. Acquiring a sufficiently large, labeled dataset can be challenging and expensive in medical imaging. As a result, researchers often use transfer learning, which involves fine-tuning pre-trained CNNs on smaller breast cancer datasets to improve accuracy and overcome data limitations [34,35].

One of the main advantages of CNNs in breast cancer detection is their robustness in handling local distortions and geometric variations, making them well suited for identifying complex patterns in breast tissue images. Studies show that both de novo CNNs (built and trained from scratch) and transfer learning-based CNNs are widely applied in breast cancer classification tasks. These methods enable CNNs to achieve high performance in distinguishing between benign and malignant lesions, even with limited data, by leveraging pre-trained weights and advanced feature extraction capabilities. In BC classification, de novo CNNs and CNNs based on transfer learning are widely used [36].

**Extreme Learning Machines (ELMs):** Extreme Learning Machines are a type of neural network with a single hidden layer, and are known for their high-speed learning capabilities and generalization performance. In breast cancer diagnosis, ELMs are applied to classify medical images and detect abnormalities in breast tissue with minimal training time. Unlike traditional neural networks, ELMs do not require iterative tuning of hidden nodes, making them computationally efficient and suitable for real-time applications. ELMs have been shown to perform well with smaller datasets, which is particularly useful in medical fields where labeled data may be scarce [37,38]. Studies indicate that ELMs can effectively classify benign and malignant breast lesions with competitive accuracy, though they may sometimes lack the deep feature extraction capabilities of more complex networks [39].

**Generative Adversarial Networks (GANs):** Generative Adversarial Networks are powerful tools for generating synthetic data to augment limited datasets. GANs consist of two neural networks—the generator and the discriminator—that work in tandem through a competitive training process. In the context of breast cancer, GANs can create synthetic mammography or ultrasound images that resemble real samples, enriching the training dataset and helping improve the robustness of diagnostic models [29].

This data augmentation is essential, especially when access to large, labeled datasets is constrained. GANs also facilitate domain adaptation, enabling models trained on synthetic data to generalize better when applied to real-world cases. However, GANs can be complex to train due to issues like mode collapse and instability, requiring careful parameter tuning and extensive computational resources [40,41].

**Deep Convolutional Neural Networks:** Deep convolutional neural networks (DCNNs) have shown remarkable success in breast cancer image classification by utilizing multiple layers that allow them to learn complex hierarchical features from large datasets. DCNNs are particularly effective in breast cancer diagnostics, as their depth enables them to capture subtle patterns in mammograms and histopathological images, leading to more accurate classification between benign and malignant tissues [19].

Studies indicate that lightweight CNNs designed and trained specifically for breast cancer detection can sometimes outperform conventional pre-trained models, such as those based on ImageNet. These lightweight models, trained from scratch with specific datasets related to breast cancer, demonstrate higher accuracy and efficiency for this task. For example, a study comparing lightweight CNNs with conventional transferred models found that smaller, custom-trained CNNs yielded better results in diagnosing breast cancer, likely due to their targeted feature extraction capabilities [35].

The process typically involves feeding the breast cancer images into a DCNN, which extracts relevant features through its convolutional and pooling layers and classifies the findings in fully connected output layers. This structured approach enhances the network’s ability to distinguish between different types of breast lesions, improving diagnostic accuracy and supporting clinical decision-making [34,42,43].

**Figure 2:**
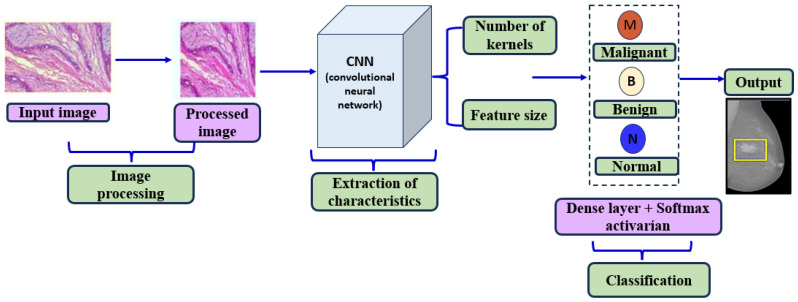
Medical image classification process using a convolutional neural network (CNN).

Figure 2 shows a diagram illustrating a medical image classification process using a convolutional neural network (CNN). The process begins with the enhancement of an input image, which undergoes image processing to improve quality and highlight key features. The processed image is then fed into the CNN, where relevant features are extracted based on the number and size of kernels in each layer. These features are passed through a dense layer with SoftMax activation, allowing the CNN to classify the image into one of the predefined categories (malignant, benign, or normal), each corresponding to different diagnoses or tissue types. The final output represents the result of this classification process, aiding in the detection of potential abnormalities in medical imaging.

**Figure 3:**
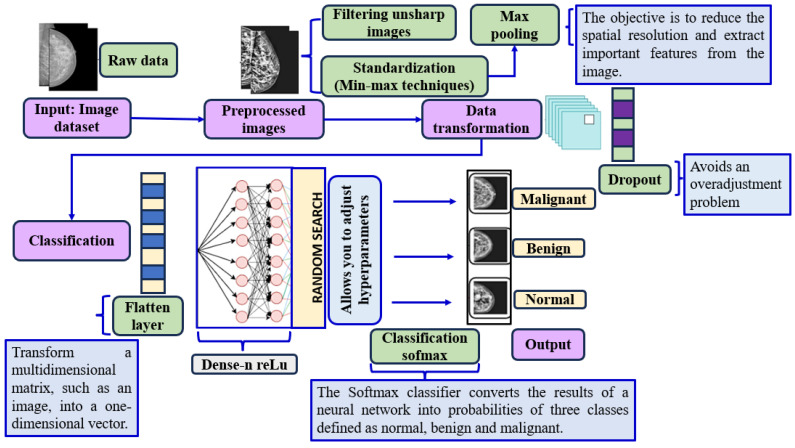
CNN-based diagnostic workflow for breast cancer image classification.

Figure 3 illustrates the workflow of an automated diagnostic system based on convolutional neural networks (CNNs) for breast cancer detection from medical images. It begins with an image dataset containing raw data, which undergoes image preprocessing, including techniques such as filtering unsharp images. Next, data transformation is performed using standardization techniques (Min-Max Techniques) and Max Pooling to reduce spatial resolution and extract important features. A dropout layer is then applied to prevent overfitting. The image is flattened into a one-dimensional vector through the flatten layer and passed through a dense layer with ReLU activation to learn complex patterns. Random Search is used to tune the hyperparameters for optimal network performance. Finally, a SoftMax layer converts the results into probabilities for each class (malignant, benign, and normal), producing an output that classifies the image into one of these three categories, aiding in the detection of potential abnormalities in mammograms.

## 4. Methodology

All studies included in this systematic review were sourced from publicly available research databases, ensuring that only accessible and verifiable studies were analyzed. The review process adhered to the PRISMA (Preferred Reporting Items for Systematic Reviews and Meta-Analyses) guidelines (Supplementary Materials), which provide a standardized approach for conducting systematic reviews. The PRISMA flow diagram and protocol were employed to systematically guide the study selection process, encompassing identification, screening, eligibility, and the inclusion of relevant studies. This framework ensures transparency and reproducibility in the study selection and data extraction processes, thereby enhancing the rigor and reliability of the review [44,45].

### 4.1. Eligibility Criteria

Studies that reported data on the diagnostic accuracy of deep learning models for breast cancer detection using mammogram images were included. These studies evaluate these models’ development and implementation in breast cancer screening programs. The selected articles were downloaded and imported into Mendeley for reference management. After removing duplicates, three researchers (J-R.P.-N., C.R., and L.-J.V.-S.) independently reviewed the titles and abstracts to assess the eligibility of each article. Discussions among the authors resolved any differences in judgment until a common agreement was reached. Searches were performed on IEEE, SCOPUS, and PubMed. The inclusion criteria were as follows: (a) deep learning, (b) breast cancer, (c) convolutional neural networks, (d) hybrid models, and (e) image patterns. The exclusion criteria were as follows: (f) age older than five years, (g) duplicates, and (h) studies related to other types of cancers.

### 4.2. Information Sources and Search Strategy

The research questions and search terms were formulated using the PICO (Participants/Population, Intervention, Comparison, Outcomes) scheme [35], focusing on women at risk of breast cancer. The intervention was defined as applying intelligent algorithms in deep learning, and comparisons were made about breast cancer risk estimation and prediction. Given the exploratory nature of the study, specific results were not included. A literature search strategy was developed to structure key terms and facilitate the retrieval of the available literature in the database. This strategy combined the main concepts of the research question to obtain the desired results. The research questions and search criteria used are presented in Table 2 and Table 3.

### 4.3. Study Selection

The PRISMA 2020 flow diagram outlines the systematic review process for breast cancer studies, detailing the identification, screening, eligibility, and inclusion phases. The systematic review process started with the identification of 4339 records from three databases: IEEE (1.5%), Scopus (87.6%), and PubMed (10.9%). Following the removal of 30 duplicates (0.7%) and 3945 irrelevant records (90.9%), 364 records (8.4%) remained for screening. During this phase, 193 records (53%) were excluded for not aligning with the research topic, leaving 171 records (47%) for full-text eligibility assessment. In this phase, 28 studies (16.4%) were excluded due to publication year, and 81 (47.4%) were excluded based on title, abstract, or conclusion. Finally, 62 studies (36.3% of the eligible records) were included in the review: 12 from IEEE (19.4%), 40 from Scopus (64.5%), and 10 from PubMed (16.1%). This meticulous process ensured the inclusion of only the most relevant studies for the systematic review.

### 4.4. Study Extraction and Synthesis

**Figure 4:**
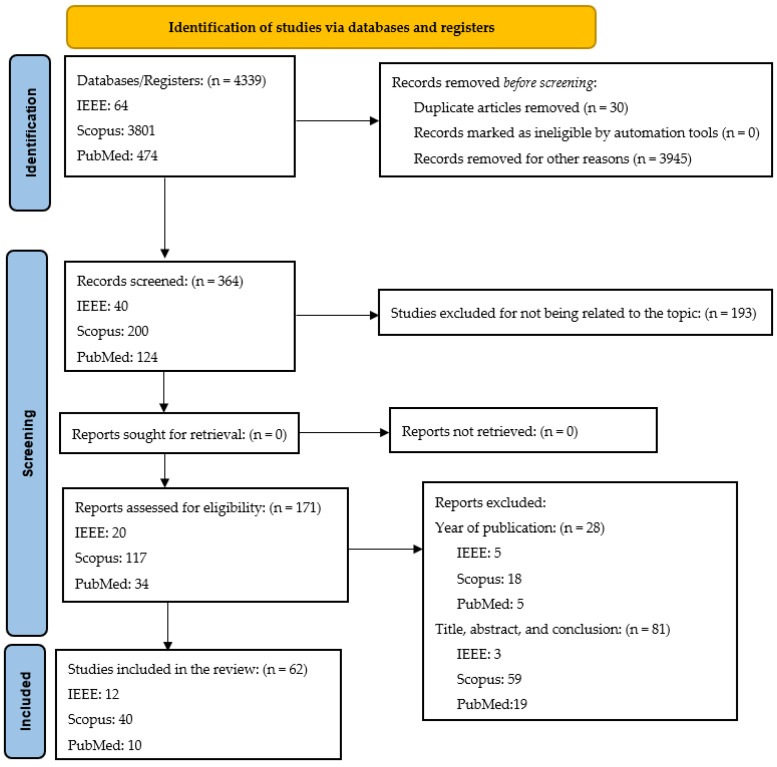
PRISMA 2020 flow diagram for systematic reviews.

Two independent investigators (J.-R.P.-N. and L.-J.V.-S.) screened each study based on the title and abstract to determine its eligibility for the full-text review stage. In addition, the reference list of the selected articles was reviewed to identify studies that might have been missed in the initial search. Any conflicts in selection were resolved by a third reviewer (C.R.). For each study, the following data were extracted: author(s), year, study design, and relevant findings (Table 4, Table 5, Table 6, Table 7 and Table 8). Data extraction was performed by one reviewer (J.-R.P.-N.), with collaboration from the other authors (L.-J.V.-S., C.R., and C.N.). Studies published in languages other than English, narrative or systematic reviews, meta-analyses, and conference proceedings were excluded due to high risk of bias (Figure 4).

## 5. Results

A summary of current research on the identification and categorization of breast cancer utilizing a range of deep learning and artificial intelligence-based techniques is shown in Table 4. The papers cover a wide range of methods and strategies, from hybrid models that combine machines and deep learning to convolutional neural networks (CNNs). Enhancing diagnostic precision, spotting subtle patterns in mammograms, and providing therapeutic decision assistance are just a few of the objectives of these investigations. Each study uses various classification techniques, including bagging algorithms, deep neural networks, and transfer learning models, to maximize the accuracy of identifying benign and malignant lesions in medical images. Reported accuracies range from 82.3% to 100%, highlighting the significant potential of these models to enhance diagnostic precision compared to conventional methods. In conclusion, this table highlights the evolution and effectiveness of artificial intelligence models in breast cancer diagnosis, highlighting their potential to transform the treatment and prognosis of this disease.

**Table 4:** Analysis of findings by selected source.

Author	Title	Objective	Classification Method	Accuracy	Dataset
Gu et al. (2022)	Deep learning based on ultrasound images assists breast lesion diagnosis in China: a multicenter diagnostic study [46]	To evaluate the accuracy of the deep learning model in detecting and classifying benign and malignant breast lesions compared with diagnoses made by expert radiologists.	CNN	86.40%	It uses 14,043 ultrasound images from multiple centers in China (32 hospitals)
Yala et al. (2019)	A deep learning mammography-based model for improved breast cancer risk prediction [23].	The model demonstrates that DL can identify subtle signals present in mammograms. Furthermore, the model automatically learns to discover these patterns from the data by using DL.	Deep Learning Hybrid Logistic Regression Model	95%	Contains 88,994 mammography images (Massachusetts General Hospital)
Gu et al. (2023)	Breast Cancer Prediction Using Fine Needle Aspiration Features and Upsampling with Supervised Machine Learning [47].	Using the SMOTE method to address the imbalance in the dataset, the influence of feature set size was analyzed using various feature and feature set selection techniques.	Random Forest, SVM, Gradient Boosting Machine, Logistic Regression, Multilayer Perceptron, and KNN	100%	Wisconsin Breast Cancer Dataset (WBCD)
Alzu’bi et al. (2021)	Predicting the recurrence of breast cancer using machine learning algorithms [48].	The bagging classifier outperformed the other classifiers used in the experiments to provide clinical decision support. The OneR classifier had the lowest error rate and the shortest time to build the model.	Bagging	92%	King Abdullah University Hospital (KAUH) Breast Cancer Recurrence Dataset
Singh et al. (2023)	The artificial intelligence-based medical decision support system is used for accurate and early breast cancer prediction [49].	The model categorizes breast cancer into two groups using the reference feature set (WDBC) and selects the fewest number of features for maximum accuracy.	Eagle Strategy + Gravitational Search Optimization (Hybrid Model)	98.95%	Wisconsin Diagnostic Breast Cancer
Sun and Li (2023)	A Study of Breast Cancer Classification Algorithms by Fusing Machine Learning and Deep Learning [50].	They design deep learning model structures to discover hidden patterns in selected features. The model was trained on a specific dataset to be efficient and low-cost.	Model Fusion Algorithm (ML Algorithm + Neural Network)	98.3%	Unspecified dataset
Asadi and Memo (2022)	Layered Deep learning for Improved Breast Cancer Detection [51].	These models detect breast cancer and help facilitate a faster expert decision on cancer status. The models were trained on the ImageNet dataset.	CNN	96%	ImageNet
Karthik et al. (2023)	Novel Deep CNN Model based Breast Cancer Classification [52].	They built a DeepCNN model to classify breast cancer more accurately from digital mammography images.	DeepCNN with Random Search Optimizer	99.18%	INBreast, mini-MIAS, mini-DDSM, MIAS, DDSM
Siddiqui et al. (2021)	IoMT Cloud-Based Intelligent Prediction of Breast Cancer Stages Empowered with Deep Learning [53].	An Internet of Medical Things (IoMT) cloud-based model for intelligent breast cancer stage prediction is proposed.	IoMT + CNN	97.81%	IRMA (355 images)
Chorianopoulos et al. (2020)	Deep Learning Methods in Medical Imaging for the Recognition of Breast Cancer [54].	Medical images, specifically ultrasound and breast histopathology images, are analyzed to assess their ability to detect breast cancer.	CNN	96.82%	Ultrasound and histopathology images (does not specify the exact hospital or country)
Lu et al. (2019)	The Classification of Mammogram Using CNN with Specific Image Preprocessing for Breast Cancer Detection [55].	Techniques such as median filtering, contrast-limited adaptive histogram equalization, and data enhancement were applied to more than 9000 mammograms. Subsequently, a classifier model was trained using a convolutional neural network.	CNN	82.3%	BI-RADS
Lupat et al. (2023)	Moanna: Multi-Omics Autoencoder-Based Neural Network Algorithm for Predicting Breast Cancer Subtypes [56].	It uses multi-omics data to predict breast cancer subtypes. Moanna overcomes the limitations of existing methods by integrating gene expression, copy number, and somatic mutation data.	Semi-Supervised Automatic Encoder	96%	METABRIC (Molecular Taxonomy of Breast Cancer International Consortium)
Selvathi and Aarthypoornila (2018)	Performance analysis of various classifiers on deep learning network for breast cancer detection [57].	A sparse automatic encoder is implemented to extract relevant features from mammography images and, subsequently, a cascade classifier is used to perform classification based on these features.	Convolutional Sparse Autoencoder (Random Forest Classifier)	98.89%	INbreast
Janney et al. (2023)	Automated Detection of Breast Cancer using Artificial Intelligence Systems [58].	Using breast histopathology images, grading distinguishes between benign and malignant tumors. K-means grouping is used to determine the precise grades of cancer.	CNN (AlexNet and GoogLeNet Architecture)	AlexNet: 100%	BreaKHis
Ashfaq et al. (2022)	Breast Cancer Diagnosing Empowered with Transfer Learning [59].	A transfer learning model using the AlexNet convolutional neural network extracts features from breast magnetic resonance imaging (MRI) and trains a breast cancer detection model.	CNN (ALexNet Architecture)	99.65%	Magnetic resonance imaging (MRI)
Asha et al. (2023)	Breast Cancer classification using Neural networks [60].	DNN, CNN, ANN, and RFE are analyzed for function selection. The study categorizes the breast cancer dataset.	DNN, ANN, CNN, and RFE	DNN:97%	It does not specify the database (dataset is taken from Kaggle: 30 feature variables and 569 occurrences)
Abbasniya et al. (2022)	Classification of Breast Tumors Based on Histopathology Images Using Deep Features and Ensemble of Gradient Boosting Methods [61].	The study develops a computer-aided diagnosis (CAD) system using deep feature transfer learning to improve the accuracy and efficiency of breast cancer diagnosis through histopathology images.	IRv2-CXL (- Feature extraction: Inception-ResNet-v2. - Classification algorithms: CatBoost, XGBoost, and LightGBM)	96.46%	BreaKHis
Li et al. (2022)	Dynamic Weight Agnostic Neural Networks and Medical Microwave Radiometry (MWR) for Breast Cancer Diagnostics [62].	The study optimizes neural network architecture and improves accuracy using a bipopular covariance matrix adaptive evolution strategy (BIPOP-CMA-ES).	Weight-Agnostic Neural Network with BIPOP-CMA-ES Weight Optimization.	93.2%	MWR (Medical Microwave Radiometry)
Maleki et al. (2023)	Breast cancer diagnosis from histopathology images using deep neural network and XGBoost [63].	The study improves the speed and accuracy of breast cancer diagnosis by using deep learning in a CAD system to classify histopathological images efficiently.	Feature extraction: DenseNet201, VGG16, VGG19. Final classifier: XGBoost	91.9%	BreakHis (7909 histopathological images)

**RQ1:** What studies exist on deep learning for preventing, automatically diagnosing, and treating breast cancer?

In this section, Table 5 summarizes various studies that have explored deep learning techniques for the prevention, automatic diagnosis, and treatment of breast cancer. These studies utilize different datasets and models, with methods ranging from pre-trained convolutional neural networks (CNNs) and stacked autoencoders to more complex architectures like YOLOv3 and Faster-RCNN. The performance metrics reported include accuracy, precision, and area under the curve (AUC), with most studies achieving high accuracy and precision rates, highlighting the effectiveness of deep learning in breast cancer detection and classification. The studies listed explore a range of deep learning methods for preventing, automatically diagnosing, and treating breast cancer. For instance, Chougrad et al. [64] used pre-trained CNNs optimized through grid search on various datasets, achieving AUC values as high as 94%. Zheng et al. [65] employed a stacked autoencoder with an AdaBoost classifier, reporting an accuracy of 97.2%. Dabass et al. [66] used a Hanman transform classifier and achieved 100% accuracy in distinguishing between benign and malignant cases. Additionally, methods like YOLOv3, used by Al-antari et al. [67], and Faster-RCNN, used by Agarwal et al. [68], demonstrated high precision and accurate positivity rates in detecting breast cancer lesions. These studies underscore the potential of deep learning to significantly enhance the accuracy and efficiency of breast cancer diagnosis and treatment.

**Table 5:** Advances in deep learning for breast cancer.

Author	Model/Method	Techniques	Performance Metrics
**1. CNN-Based Models**			
Chougrad et al. [64].	Pre-trained CNN VGG with grid search optimization.	Learning rate, epochs, dropout rate, weight decay, momentum.	AUC: 86% (MIAS) AUC: 89% (DDSM) AUC: 93% (INBreast) AUC: 94% (BCDR)
Al-antari et al. [67].	Detecting lesions with YOLOv3; routine feedback CNNs for classification include ResNet-50 and Inception ResNet-V2.	YOLOv3 for precision detection.	Precision: 99.17% (DDSM) Precision: 97.27% (INBreast)
Al Fryan et al. [69].	CNN (AlexNet, VGGNet, Lenet, GoogLeNet) and proposed model with wider parallel kernels.	Image normalization based on H&E staining. Mask R-CNN for nuclear segmentation. K-means clustering for ROI extraction.	Accuracy: 79% (Top 1: TCGA) Accuracy: 94.5% (Top 5: TCGA) AUC: 85% (METABRIC) Accuracy: 78.5% (BTH) Sensitivity: 83% (BTH) Specificity: 74% (BTH)
**2. Autoencoder-Based Models**			
Zheng et al. [65]	Stacked autoencoder with AdaBoost classifier	Unsupervised learning, deep convolutional network.	Accuracy: 97.2% (not specified)
**3. Transform and Feature Extraction Methods**			
Dabass et al. [66]	Hanman transform classifier (HHT) with dithering.	Energy features, sigmoid features, practical information.	Accuracy: 100% (MIAS: benign vs. malignant)
Sebastien Jean Mambou et al. [70]	Pre-trained Inception V3 + SVM.	Transfer learning, ROI extraction, grayscale preprocessing and feature classification, and inner-product layers.	Accuracy: 79% (Top-1: DMR) Accuracy: 94.5% (Top-5: DMR) Sensitivity: 78% (For women under 50 years) Sensitivity: 78–89% (For women < 50)
**4. Faster-RCNN and Region Proposal Networks (RPNs)**			
Agarwal et al. [68]	Faster-RCNN with InceptionV2 for mass detection	RPN feature maps, CNN feature extraction.	TPR: (91 ± 6)% (GE scanner) TPR: (99 ± 3)% (INBreast- Malignant) TPR: (85 ± 8)% (INBreast- Benign)
**5. Optimized Network Architectures**			
L. Shen et al. [71]	Deep disproved network with improved sunflower optimization.	Feature classification.	Accuracy: 91.5% (MIAS)
Stepan Romanov et al. [72].	MAI-risk (Manchester Artificial Intelligence risk model).	Pre-trained ResNet-18 for feature extraction. Attention-based pooling for feature aggregation. Image patching (224 × 224). Data normalization and augmentation (Otsu’s segmentation, affine transforms).	AUC: 74.7% (PROCAS) AUC: Screen-detected cancers 67.6%. (PRC-**mixed**) AUC: 74.7% (BCR-**priors**)
**6. Segmentation and Fuzzy Classification Techniques**			
T. Shen et al. [73]	ResU-segNet with hierarchical fuzzy classifier (HFC).	Segmentation, fuzzy c-means in the interval type-2 and fuzzy neural networks.	AUC: 93.26% (INBreast) AUC: 90.67% (private datasets)

**RQ2:** What are the benefits and disadvantages of the various methods employed?

Several methods were applied at different stages. For the final sorting, RF, SVM, and ANN were chosen. Highlighting: In the medical imaging field, the deep learning CNN model was distinguished for its exceptional abilities in automated feature extraction. Pre-training of the model, which saves time during training, contributes to CNNs’ popularity. In addition, U-Net was employed to identify the affected breast areas and segment the image accurately. Evolutionary methods like swarm algorithm, Grasshopper optimization, and GA were used to optimize the analysis in some situations.

**Table 6:** Methods to see their advantages and disadvantages.

Author	Method	Advantages	Limitations
Pérez-Benito et al. (2020)	Fully convolutional-based neural network (ECNN) [74].	Performance is the same depending on the radiologist’s opinion.	Other mask-based, supervised, and unsupervised methods need to be investigated.
George et al. (2019)	NucTraL + BCF: transfer learning + Kernel patches + belief theory-based classifier fusion [75].	Cost-effective and low complexity.	The results of the proposed method should be provided for a lower magnification scale (40 × 100).
Chang et al. (2020)	Evaluation of crowdsourcing [76].	Crowdsourcing is adequate for evaluating model performance.	When the model is examined using data formats other than the trained one, its performance deteriorates.
Yap et al. (2020)	The fastest 3-channel artificial RGB learning transfer: RCNN with Incorporation ResNet-v2 [77].	FCN-AlexNet works well for an extensive heterogeneous dataset. Faster-RCNN is more efficient when assessed within an individual dataset.	The differences between the two networks might be overestimated.
Singh et al. (2020)	In addition to VGG-19, supplemented with deep layers, logistic regression, random forest, and SVM [78].	Rank unbalanced datasets in multiple ways.	No method was successful on every evaluation criterion.
Singh et al. (2020)	Capture spatial and scale context: Atrous convolution + Promote the tumor-relevant features: Propose the use of channel attention along with channel weighting. (CAW) + Capture local context information: Integrate the structural similarity index metric (SSIM) and L1 norm [79].	Improves diagnostic efficiency for healthcare professionals. IoU (intersection area and union area between predictions) and Dice scores (metric used to assess the similarity between two sets) of 88.82% were achieved compared to six segmentation methods.	The accuracy of automatic segmentation can be affected by low signal quality and noise in ultrasound images. The model’s applicability is limited by the quality and quantity of training data; the fact that tumors vary in size and shape makes accurate segmentation difficult and less effective.
Moon et al. (2020)	The suggested CNN-based method includes VGGNet, ResNet, and DenseNet [80].	Using images from various ultrasound machines and reducing errors through ensemble techniques and diverse CNN architectures improves model performance.	Not considering surrounding tissue, the risk of overfitting, and reliance on manual ROI definition can affect diagnostic accuracy.
Shu et al. (2020)	Region-based Group-Max Pooling (RGP) and Global Group-Max Pooling (GGP) [81].	Approximately located suspicious regions.	Test model improvement.
Yari et al. (2020)	Implementation of DenseNet121 and ResNet50 models for medical diagnostics [82].	Evaluating nine different models and optimized hyperparameters.	BreakHis dataset is small (82 patients), and accuracy decreases at 400× images.
Bhowmik and Eskreis-Winkler (2022)	Deep learning (DL), Segmentación Automática, Modelos Predictivos, Redes Convolucionales (CNN), transfer learning, Integración Multimodal [83].	Enhances diagnostic accuracy, automates tasks, reduces unnecessary biopsies, identifies complex patterns, supports real-time decision-making, and enables personalized medicine.	Limited generalizability due to retrospective studies, reliance on large, diverse datasets, “black box” nature, and variability in imaging protocols.
Zhou et al. (2020)	ROI-based, Radiomics, and deep learning (ResNet50) [84].	High diagnostic accuracy with the smallest bounding box (accuracy: 91%; testing: 89%) and peritumor tissue improves accuracy.	It relies on tumor segmentation quality, a small dataset for deep learning, and challenges in applying models to non-mass-like lesions.

**RQ3:** Which methods and metrics showed the greatest efficiency?

In recent years, numerous deep learning methods have been developed and refined for the prevention, automatic diagnosis, and treatment of breast cancer, aiming to enhance the accuracy and efficiency of these processes. The following studies demonstrate some of the most effective models, achieving high performance metrics across various datasets. For instance, Ribli et al. utilized Faster-RCNN on the INBreast and Digital Mammography DREAM Challenge datasets, achieving an AUC of 95% and 85%, respectively. Yu et al. applied multiple models, such as GoogleNet and AlexNet, on the BCDR dataset, reporting AUC values ranging from 60% to 81%. Other notable methods include Ting et al.’s CNNI improvement on the MIAS dataset, which achieved a sensitivity of 89.47% and an accuracy of 90.50%, and Vijayarajeswari et al.’s Hough transform with SVM, reaching an accuracy of 94%. Additionally, models like Dense-Net-II, VGG Net, and DenseNet, applied by Li et al. on digital mammograms, yielded accuracies above 92%, with DenseNet-II reaching 94.55%. Cutting-edge methods such as the LFR-COA-DenseNet121-BC model used by Emam et al. achieved near-perfect accuracy, sensitivity, and specificity, emphasizing the rapid advancements in this field. In addition, several metrics are used in Table 7 to evaluate the performance of the models. Among these metrics are the confusion matrix, precision, recall (also known as sensitivity or true positive rate), specificity, false positive rate (FPR), F1-score, area under the curve (AUC), and Jaccard index (Jaccard index).

**Table 7:** Methods that produced higher accuracy.

**Digital Mammography**			
**Author**	**Dataset**	**Method**	**Efficiency**
Ribli et al. (2018)	Digital mammography DREAM Challenge [85].	Faster-RCNN	AUC: 85%
	INBreast [85].		AUC: 95%
Yu et al. (2019)	BCDR [86].	GoogleNet	AUC: 88% Accuracy: 81%
		AlexNet	AUC: 83% Accuracy: 79%
		CNN3	AUC: 82% Accuracy: 73%
		CNN2	Accuracy: 72%
		GLCM	Accuracy: 69%
		Hu’s moments	Accuracy: 62%
Ting et al. (2019)	MIAS [87,88].	CNNI (convolution neural network improvement)	Sensitivity: 89.47% Accuracy: 90.50% AUC: (90.1 ± 3.14)% Specificity: 90.71%
Vijayarajeswari et al. (2019)		Hough transform + SVM	Accuracy: 94%
Li et al. (2019)	Digital mammograms were obtained from the First Hospital of Shanxi Medical University [89].	DenseNet-II	Accuracy: 94.55% Sensitivity: 95.60% Specificity: 95.36%
		VGG Net	Accuracy: 92.78% Sensitivity: 93.58% Specificity: 92.42%
		DenseNet	Accuracy: 93.87% Sensitivity: 94.59% Specificity: 93.90%
		GoogleNet	Accuracy: 93.54% Sensitivity: 93.90% Specificity: 93.17%
		AlexNet	Accuracy: 92.70% Sensitivity: 93.60% Specificity: 91.78%
Hossain (2022)	DDSM [90,91].	Detect suspicious regions: Fuzzy C-means clustering algorithm and train U-net segmentation network	Jaccard index: 97.4% Accuracy: 98.2% Sensitivity: 98.4% Precision: 94.7% F-score: 97.8%
Song et al. (2020)		DCNN	Accuracy: 80.38%
		Score-XGBoost	Accuracy: 81.92%
		Feature-XGBoost	Accuracy: 80.38%
		Score Feature-XGBoost	Accuracy: 84.48%
Yu et al. (2021)	MINI-MIAS and INbreast [92,93].	CNN: DisepNet	Accuracy: 95.60% Sensitivity: 93.71% Specificity: 97.44%
Zhang et al. (2021)		BDR-CNN-GCN	Accuracy: 96.10 ± 1.60% Sensitivity: 96.20 ± 2.90% Specificity: 96.00 ± 2.31% Precision: 96.06 ± 2.14% F-score: 96.10 ± 1.61%
**Biopsy Images**			
**Author**	**Dataset**	**Method**	**Efficiency**
Li et al. (2019)	Biopsy images: Israel Institute of Technology (IEIT) [94].	Fully convolutional autoencoder + SVM + one-layer neural network	F1-score: 77.7% Accuracy: 76%
Bejnordi et al. (2019)	Dataset of 2387 biopsy whole-slide images from 882 patients (BREAST) [95].	Deep convolutional neural networks (CNNs)	AUC: 96.2% (For classifying invasive carcinoma vs. benign biopsies)
		Stacked architecture with three CNNs (networks I, II, and III). Focus on tumor-associated stroma analysis	Accuracy: 95.5% (pixel-level classification—network I).
**Infrared Images**			
**Author**	**Dataset**	**Method**	**Efficiency**
Emam et al. (2023)	DMR (infrared images) [96].	DenseNet121	Accuracy: 94.33% Sensitivity: 94.22% Specificity: 94.25% Precision 94.19% F-score 94.23%
		LFR-COA-DenseNet121-BC	Accuracy: 99.97% Sensitivity: 99.96% Specificity: 99.95% Precision: 99.96% F-score: 99.96%
**Ultrasound Images**			
**Author**	**Dataset**	**Method**	**Efficiency**
Shu Zama et al. [97].	1008 ultrasound images. (Histological types: 202 cysts, 203 fibroadenomas, 201 scirrhous IDCs, 200 solid IDCs, 202 tubule-forming IDCs)	DCGAN (deep convolutional GAN): Generates synthetic images for training and evaluation Image synthesis performed over 200 epochs	**Benign vs. Malignant** Accuracy: 100% (Reader 1, synthetic) Accuracy: 94% (Reader 1, original) Accuracy: 100% (Reader 2, synthetic) Accuracy: 96% (Reader 2, original) **Histological type** Accuracy: 86% (Reader 1, synthetic) Accuracy: 78% (Reader 2, synthetic)
**Histopathological Images**			
**Author**	**Dataset**	**Method**	**Efficiency**
Luz et al. (2022)	Histopathological images [98].	VGG-19	Accuracy: 86.80% AUC: 95.34%
		ResNet-50	Accuracy: 83.38% AUC: 84.35%
		Xception	Accuracy: 82% AUC: 84.73%
		DenseNet-121	Accuracy: 83.57% AUC: 86.26%
		DenseNet-201	Accuracy: 83.74% AUC: 93.88%
		Inception-V3	Accuracy: 81.90% AUC: 92.03%
Vaka et al. (2020)	Histopathology image taken from M. G Cancer Hospital & Research Institute, Visakhapatnam, India [99].	NB	Accuracy: 95.61% Precision: 95.65% Recall: 93.61%
		SVM	Accuracy: 95.61% Precision: 95.65% Recall: 93.61%
		Bi-clustering and AdaBoost techniques	Accuracy: 95.75% Precision: 95.72% Recall: 96.26%
		RCNN	Accuracy: 91.3% Precision: 91.3% Recall: 89.3%
		HA-BiRNN: bidirectional recurrent neural network	Accuracy: 82.50% Precision: 80.09% Recall: 79.03%
		DNNS: deep neural network with support value	Accuracy: 97.21% Precision: 97.9% Recall: 97.01%
Kollem et al. (2023)	BreakHis and BreCaHAD [100].	PResNet-34 + FE-VGG-16 + M-AlexNet (with augmentation): **DT**	Accuracy: 98.99% Sensitivity: 98.72% Specificity: 97.89% Precision: 97.87% F1-score: 97.76%
		PResNet-34 + FE-VGG-16 + M-AlexNet (with augmentation): **KNN**	Accuracy: 97.34% Sensitivity: 97.14% Specificity: 97.31% Precision: 97.34% F1-score: 97.11%
		PResNet-34 + FE-VGG-16 + M-AlexNet (with augmentation): **LDA**	Accuracy: 96.18% Sensitivity: 96.59% Specificity: 96.88% Precision: 96.67% F1-score: 96.81%
		PResNet-34 + FE-VGG-16 + M-AlexNet (with augmentation): **LR**	Accuracy: 95.18% Sensitivity: 96.04% Specificity: 96.35% Precision: 96.53% F1-score: 96.12%
		PResNet-34 + FE-VGG-16 + M-AlexNet (with augmentation): **SVM**	Accuracy: 99.99% Sensitivity: 99.56% Specificity: 99.53% Precision: 99.55% F1-score: 99.77%
		PResNet-34 + FE-VGG-16 + M-AlexNet (without augmentation): **DT**	Accuracy: 96.95% Sensitivity: 95.54% Specificity: 96.87% Precision: 96.66% F1-score: 96.65%
		PResNet-34 + FE-VGG-16 + M-AlexNet (without augmentation): **KNN**	Accuracy: 95.29% Sensitivity: 95.02% Specificity: 95.98% Precision: 96.13% F1-score: 95.10%
		PResNet-34 + FE-VGG-16 + M-AlexNet (without augmentation): **LDA**	Accuracy: 94.97% Sensitivity: 94.52% Specificity: 94.56% Precision: 95.96% F1-score: 94.07%
		PResNet-34 + FE-VGG-16 + M-AlexNet (without augmentation): **LR**	Accuracy: 93.97% Sensitivity: 93.82% Specificity: 93.03% Precision: 94.32% F1-score: 93.07%
		PResNet-34 + FE-VGG-16 + M-AlexNet (without augmentation): **SVM**	Accuracy: 98.99% Sensitivity: 97.31% Specificity: 97.21% Precision: 97.89% F1-score: 97.11%

**Figure 5:**
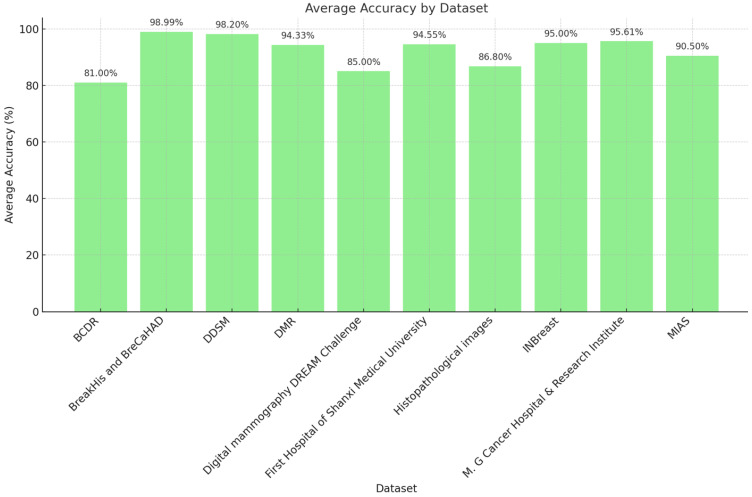
Average accuracy comparison by dataset in cancer detection methods.

Figure 5 displays a bar chart comparing the average accuracy of various detection methods applied to different medical image datasets for cancer-related pattern identification. Each bar represents a specific dataset, with the corresponding average accuracy shown above in percentages. The horizontal axis lists the dataset names, while the vertical axis represents the average accuracy in percentages.

**RQ4:** What are the most prevalent image datasets for breast cancer diagnosis?

Table 8 provides a concise overview of various research studies on breast cancer diagnosis using mammography images, highlighting different machine learning and deep learning methods across several prevalent image datasets. The studies explore techniques like transfer learning (ResNet), ResNet18 optimization, UNet for segmentation, and various classifiers such as SVM and ensemble methods. The performance metrics, including AUC and precision, demonstrate the effectiveness of these methods, with some models achieving precision as high as 98.26% (ResNet18 optimization on INBreast) and AUC values up to 99.88% (UNet on INBreast). The most prevalent image datasets for breast cancer diagnosis mentioned in the table are MIAS (Mammographic Image Analysis Society), DDSM (Digital Database for Screening Mammography), and INBreast. These datasets are widely used in various research studies to develop and evaluate models to improve breast cancer detection and diagnosis, underlining the importance of selecting the appropriate method and dataset to enhance diagnostic accuracy.

**Table 8:** Datasets available.

Category	Dataset	Method	Researchers	Results
Mammography	MIAS	Transfer Learning (ResNet) [101].	El Houby and Yassin (2021)	AUC: 94%
	DDSM			AUC: 92%
	INBreast			AUC: 94%
	MIAS	ResNet18 Optimization [102].	Sannasi Chakravarthy and Rajaguru (2022)	Precision: 98.13%
	DDSM			Precision: 97.19%
	INBreast			Precision: 98.26%
	DDSM	UNet for Segmentation [103].	Soulami et al. (2021)	AUC: 90.50%
	INBreast			AUC: 99.88%
	MIAS	Transfer Learning (VGG16, VGG19, Inception, ResNet) [104].	Saber et al. (2021)	Precision: 98.96% (VGG16)
	Mini MIAS	Multilevel Thresholding [105].	Kavitha et al. (2021)	Precision: 98.50%
	DDSM			Precision: 97.55%
	BCDR	SVM, Ensemble Classifier [106].	Chouhan et al. (2021)	AUC: 84.7% (SVM) AUC: 84.6% (Ensemble)

**Figure 6:**
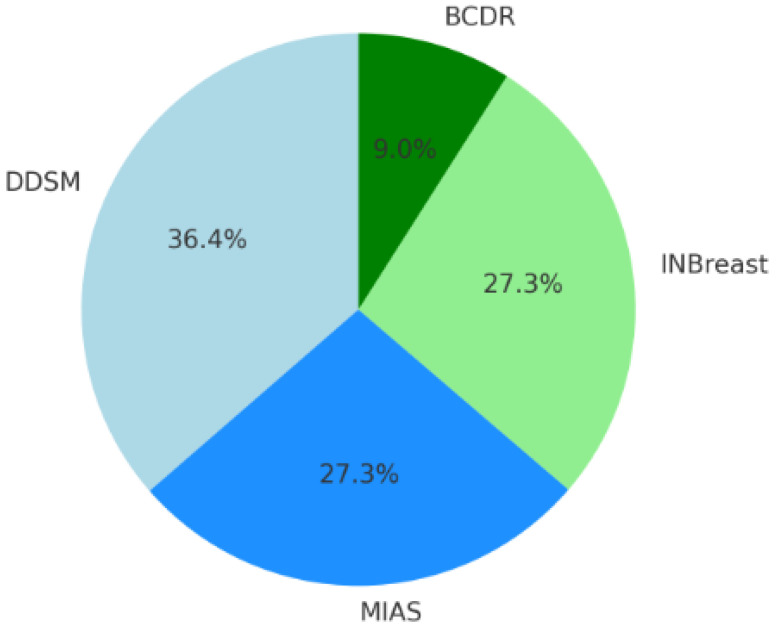
Frequency of breast cancer image dataset usage in research studies.

Figure 6 shows a pie chart that shows how frequently various breast cancer imaging datasets are used in research projects. The graph indicates that, with 36.4% of the dataset usage, the DDSM (Digital Database for Screening Mammography) dataset is the most popular. The INBreast and MIAS (Mammographic Image Analysis Society) datasets are used equally, making up 27.3% of the overall consumption. With a 9% share, the BCDR dataset is the least used. According to this distribution, the dataset used most often for breast cancer research is DDSM, followed by MIAS and INBreast. BCDR is the least widely used dataset.

## 6. Discussion

The use of deep learning (DL) methods for breast cancer diagnosis represents a significant advance in modern medicine, and their widespread adoption is becoming more widespread due to their clear advantages over traditional techniques. Despite some disadvantages, DL’s strengths in image classification far outweigh the challenges, offering transformative potential for early and accurate cancer detection.

One of the main benefits of deep learning (DL) methods in breast cancer diagnosis is their high accuracy in image classification. Several studies have shown that convolutional neural networks (CNNs) can achieve accuracy levels of over 97%, indicating a remarkable ability to distinguish between benign and malignant cancer. This high accuracy is crucial in medical diagnosis, where an error can have serious consequences. The ability of DL methods to meet and exceed these accuracy thresholds enables more reliable diagnosis, reducing the risk of false positives and negatives.

The results of this systematic review indicate that deep learning models, particularly convolutional neural networks (CNNs), demonstrate exceptional accuracy in breast cancer detection. Some architectures, such as pre-trained VGG, achieved an AUC of up to 94% on the BCDR dataset, while advanced algorithms like YOLOv3 and Faster-RCNN achieved accuracies of 99.17% and 97.27% on the DDSM and INBreast datasets, respectively, showcasing superior performance in classifying malignant lesions. Additionally, the Hanman transform classifier achieved a 100% accuracy rate on the MIAS dataset, underscoring the robustness of integrating deep learning with optimization techniques. Models leveraging transfer learning, such as AlexNet applied to magnetic resonance imaging (MRI), reached accuracies as high as 99.65%, highlighting that transfer learning enables high diagnostic precision even with limited computational resources and data availability. These findings underscore that advanced deep learning techniques enhance diagnostic accuracy and efficiency in medical image processing and mitigate the dependency on extensive labeled datasets.

In addition, deep learning technology has demonstrated a remarkable ability to improve continuously by incorporating advanced techniques such as preprocessing, feature extraction, and model fusion. These techniques improve system performance and allow for more effective adaptation to variations in the data and more detailed image analysis. This flexibility and adaptability ensures that DL models remain relevant and effective as techniques advance and new data are incorporated.

Another significant advantage of Deep learning is its ability to handle large volumes of data. Deep learning methods are especially effective at processing and analyzing large sets of medical images, an essential capability given the exponential growth of medical data. This ability enables DL systems to learn complex patterns that may not be apparent to human radiologists, improving diagnostic accuracy and processing speed.

Despite their advantages, DL methods have some disadvantages. One of the main ones is their dependence on high-quality data. To train effective models, well-labeled, high-quality data are required. The presence of noisy or poorly labeled data can negatively affect model accuracy. However, this disadvantage can be mitigated by investments in improving data collection and labeling processes, making this limitation less critical in the long run.

Another disadvantage is the need for high computational resources. DL modes, especially the deeper and more complex ones, require a considerable number of computational resources and time for training. While this need can be high, continued advances in hardware and the availability of cloud services are lowering these barriers. Significant benefits can justify investment in these resources in terms of diagnostic accuracy and efficiency.

Finally, the complexity of interpreting results is another concern. The “black box” of deep neural networks can make it difficult to understand how decisions are made, hindering validation and confidence in the results. Despite this, methods are being developed to improve the interpretability of DL models, which should increase confidence in diagnostics based on this technology as these methods evolve.

## 7. Limitations

One of the main limitations of this systematic review is the heterogeneity of the included studies, which encompass different deep learning models and approaches for breast cancer diagnosis. This makes direct comparison and synthesis of the results challenging, as reflected in the absence of a meta-analysis that could more accurately quantify the effectiveness of the methods reviewed. Additionally, there is no detailed assessment of the risk of bias in the selected studies, which could have impacted the validity of the conclusions. Another aspect to consider is the limited use of automation tools during the study selection and data extraction processes, which could have minimized potential biases and human errors. Finally, although PRISMA methodology principles were followed, there was no cross-verification between reviewers, which could have improved consistency in the interpretation of the studies.

## 8. Conclusions

The rate of mortality associated with breast cancer has decreased because of the adoption of technology. A variety of visualization techniques are employed to diagnose this disease. Deep learning (DL) techniques make lesion classification and detection easier. In addition to presenting their results according to the imaging modality, this paper provided a thorough description of the various methods of preprocessing, feature extraction, and classification that have been employed in a variety of studies.

The review highlights that convolutional neural network (CNN) architectures, like AlexNet and GoogLeNet, achieve high accuracy in classifying breast cancer, often reaching up to 100% when using transfer learning. However, other architectures, such as YOLOv3 and Faster-RCNN, have also shown remarkable performance in terms of accuracy and sensitivity, especially in malignant lesion detection tasks on datasets such as INBreast and DDSM.

The article examined and assessed the methods in terms of their pros and cons. A thorough analysis was also included to address the comprehensive review of the combined approaches. Related datasets were examined to carry out an appropriate analysis of the techniques and their outcomes. For analysis, most studies used datasets like DDSM and MIAS; they produced better results than other mammography datasets. According to the findings, identifying patterns and parameter selection can be advantageous when combining ML and DL approaches with optimization techniques. Convolutional neuronal networks (CNNs) and their variants were shown to be effective for heterogeneous datasets and when evaluated individually. It is suggested that future research should focus on multiclass classification studies to determine disease risk. Addressing problems such as overfitting and imbalance in datasets is also imperative. Therefore, it is proposed that the future framework be developed with multiclass classification in mind, incorporating ML and DL model fusion, and be automated to address problems of imbalance and overfitting.

Based on the evaluation of several studies on deep learning methods applied to breast cancer diagnosis, it is concluded that, although significant progress has been made in validation metrics such as AUC and accuracy, significant challenges affect direct comparability between results. These challenges include variability in preprocessing techniques, the heterogeneous amount of data used, and the lack of standardization in experimental evaluation. In addition, the limited availability of datasets and lack of longitudinal comparisons underscore the need for more precise and consistent standards in future research to improve the reproducibility and reliability of the models developed.

## Figures and Tables

**Table 1:** International recommendations for breast cancer screening modalities.

Population of Women	American Cancer Society	International Agency for Research on Cancer	American College of Physicians	American Academy of Family Physicians
**From 40 to 44 years old, average risk.**	Annual mammography	There is no specific recommendation	Biannual mammography if requested by the patient	Mammography if requested by the patient
**From 45 to 54 years old, average risk.**	Annual mammography	Annual mammography	There is no specific recommendation	Biannual mammography
**From 55 years and older, the average risk**	Biannual mammography, or annual if preferred	Annual mammography	Biannual mammography	Biannual mammography
**Dense breasts.**	Ultrasound or magnetic resonance imaging	There is no specific recommendation	There is no recommendation	There is no specific recommendation
**High risk.**	Annual mammography and magnetic resonance	Mammography and MRI should begin at an early age	There is no specific recommendation	There is no specific recommendation

Source: self-constructed from [18,19,20].

**Table 2:** Research questions.

Research Questions	Objective
**RQ1:** What studies exist in deep learning for preventing, automatically diagnosing, and treating breast cancer?	To explore the latest technologies and approaches in deep learning to improve accuracy and efficiency in breast cancer diagnosis.
**RQ2:** What are the benefits and disadvantages of the various methods employed?	Critically evaluate the strengths and weaknesses of deep learning methods applied to breast cancer, considering aspects such as accuracy, interpretability, generalizability, and clinical applicability.
**RQ3:** Which methods and metrics showed the greatest efficiency?	Identify and analyze specific deep learning methods that have demonstrated improved accuracy in breast cancer diagnosis or prediction, highlighting success stories and their distinguishing features.
**RQ4:** What are the most prevalent image datasets for breast cancer diagnosis?	Using deep learning, study and catalog the image datasets most frequently used in studies concerning breast cancer diagnosis.

**Table 3:** Search criteria.

Word	Criteria
**Search string**	TITLE ((“Diagnostic” OR “Identification”) AND (“breast cancer” OR “Tumour in the breast”) AND (“Deep learning” OR “Artificial Intelligence”)).
**Limits**	Academic journals (peer-reviewed); Publication date: 2018–2023; Language: English; Field of study: Computer Science, Engineering and Mathematics; Filtering by keyword: Breast Cancer and Deep learning.
**Enlarge**	Apply to equivalent words.

## Data Availability

No processing data have been generated; only a literature review has been performed.

## Supplementary Materials

The following supporting information can be downloaded at: https://www.mdpi.com/article/10.3390/diagnostics14242896/s1, PRISMA 2020 Checklist. Reference [107] is cited in the Supplementary Materials.
